# Supplementary material for: Are carbon emissions trading and green financial instruments synergistic? -Comprehensive quantitative research based on content analysis
Source: PLoS One. 2024 Mar 7;19(3):e0298601. doi: 10.1371/journal.pone.0298601 (PMC10919601; doi:10.1371/journal.pone.0298601)
Supplement: S1 Data — (ZIP) [file pone.0298601.s001.zip › Data/地级市Z_F工作报告.zip!/╝¬┴╓/═¿╗»╩╨/└┤╫╘╣1⁄2╓┌║┼í╛┬φ┐╦╩2╛▌═°í┐_2006.docx]

各位代表： 我代表市政府，向大会报告工作，请予审议。

　　一、“十五”和2005年工作简要回顾

　　过去的五年，市政府在市委的领导下，在市人大、市政协的监督支持下，抓发展、抓重点、抓落实，经过全市人民的共同努力，全面完成了“十五”计划，经济社会发展取得显著成就。

　　国民经济持续快速发展。预计2005年实现地区生产总值247亿元，比2000年增长73.6%，年均增长11.7%。人均GDP突破1万元。财政收入实现24亿元，比2000年翻了一番。粮食产量达到148万吨，畜牧业、特产业、林业和农业产业化迅速发展，农业和农村经济结构进一步优化。72种农产品通过国家绿色标识认证。规模以上工业实现增加值77.6亿元，比2000年增长87.9%，年均增长13.4%；累计实现利润59.6亿元，是“九五”的2.5倍。冶金、医药、食品三个产业增加值达到全市工业增加值的82.2%。社会消费品零售总额年均增长11.5%，外贸进出口总额年均增长32.6%。旅游收入是“九五”的7.4倍。信息产业产值年均增长24.5%。

　　振兴通化老工业基地的各项工作全面推进。制定并组织实施了《振兴通化老工业基地规划纲要》。国企改革迈出重大步伐，国有工业企业基本完成改制任务，商贸、粮食、供销、物资、社会事业以及二轻企业的改革逐步深化。股份有限公司发展到83户，有限责任公司发展到970户。组建企业集团16户。规模以上工业企业达到250户。贯彻落实中央“两免三补”惠农政策，农民人均政策性增收173.4元。加大投入力度，固定资产投资累计完成293.7亿元，是“九五”的3.2倍，其中技改投资90.3亿元，比“九五”增长3.5倍，一些关系长远发展的重点项目建成或启动。71户制药企业、187条生产线通过GMP认证。争取老工业基地国债项目和高技术专项23项，项目总数和投资额分别占全省的14.3%和22.1%。着力改善投资环境，引进各类资金309亿元，新批外商投资企业63户。推动全民创业，民营经济占GDP的比重达到58%，对财政的贡献率达到45%。

　　基础设施建设成效显著。全市新增公路里程2130公里，通梅、通五一级公路和五女峰隧道建成通车。修建农村水泥路5758公里，在全省率先实现了村村通水泥路。县城以上城市防洪达到50年一遇标准，建成农村人畜饮水工程152处，桃源水利枢纽工程竣工，国家大型商品粮基地建设项目进展顺利。完善了市县两级城市总体规划，加强了乡镇、村屯规划建设。市区累计完成城建重点工程40余项，主要街巷路基本实现硬覆盖，自来水普及率达到91.3%，集中供热面积530万平方米，煤气气化率31%，新增绿地面积13万平方米，人均公共绿地面积8.28平方米。“蓝天工程”全面完成，重点污染源得到治理，空气质量二级以上天数超过280天。城市管理得到加强，城乡环境综合整治和公路沿线治理整顿扎实推进。启动了生态市建设，植树造林、封山育林、退耕还林、打击毁林犯罪、国土资源规划保护取得阶段性成果。连续51年无重大森林火灾。

　　社会各项事业协调发展。取得市级以上科技成果56项，高新技术企业发展到39户，创建国家和省级技术研发中心10个。基础教育、职业教育较快发展，农村教育得到加强，素质教育不断推进。改造薄弱校430所，新建校舍15万平方米。集安高句丽遗迹列入世界文化遗产。社区文化、校园文化、广场文化日益活跃，推出了《长白英魂》等文艺精品力作。广播、电视覆盖率分别达到94.5%和95.6%。加强了医疗卫生服务、疾病预防控制工作，市县疾控中心等公共卫生设施投入使用。动物疫病防控体系初步形成。群众性体育活动广泛开展，竞技体育获省级以上金牌485枚。人口自然增长率下降到3.41‰。完成118个社区基础设施建设任务，社区组织和服务功能进一步完善。开展了“通化山城是我家，我为山城添光彩”等群众性精神文明创建活动。荣获全国双拥模范城“五连冠”。妇女儿童、老龄和残疾人事业不断发展。外事、侨务、民族、宗教等工作扎实推进。人防、气象、地震监测、史志、红十字会等工作取得新的成果。

　　人民生活进一步改善。城镇居民人均可支配收入达到8807元，农民人均纯收入达到3326元，分别比2000年增加4416元和673元。城镇人均住房面积增至25平方米，农村人均住房面积增至22平方米。农村“新居工程”累计建房195万平方米，改善了2.97万户农民的居住条件。归集住房公积金10.8亿元。城镇新增就业12万人。以养老、失业、医疗保险为重点的社会保障体系日趋完善。城市享受低保的人数达到10.9万人，实现了应保尽保和分类施保。农村低保覆盖面扩大到6%，享受低保待遇的达到7.2万人。城乡社会救助体系初步形成，救助困难群众20万人次。联乡包村万户脱贫工程取得积极进展。

　　民主法制和政府自身建设不断加强。主动接受人大法律监督、工作监督和政协民主监督。办理人大代表建议925件、政协提案882件，办复率100%。完成了“四五”普法任务。认真处理群众来信来访，进一步加强安全生产工作，深入开展严打整治斗争，维护了社会稳定。食品药品“放心工程”和葡萄酒行业整顿取得阶段性成果。实行市民旁听、人民建议征集、决策听证、专家咨询、社会公示制度，深入推行政务公开，大力发展电子政务，增强了政府工作的透明度。认真贯彻实施《行政许可法》，全面清理了非行政许可项目，行政审批事项削减68.8%。实施了政府机构改革。加大软环境整治和党风廉政建设工作力度，纠正部门和行业不正之风收到良好成效。

　　2005年是在振兴通化老工业基地取得良好开局基础上实现加快发展的一年。地区生产总值比上年增长14.8%，财政收入增长20%，规模以上工业增加值增长27.9%。固定资产投资翻了一番，增速居全省第一位。项目建设、国企改革、招商引资、全民创业、环境建设等重点工作取得突破性进展。全市在建项目数量增长1.2倍，新建项目增长2.1倍，开工建设亿元以上重点项目63项。热轧项目投产，冷轧项目开工，通沈高速公路开始建设，热电、人胰岛素、市区绕越线公路等重点项目进展顺利。列入省市计划的71户国有工业企业基本完成改革任务。有27户非工业国有企业完成改制。妥善安置职工6.3万人，化解金融债务22.9亿元。通钢分离办社会职能工作全面完成。开展了“创业促就业”、“万户民企进社保”等活动。城镇新增就业5.8万人。养老和失业保险参保人数分别达到22.5万人和17.6万人。社保试点“并轨”任务基本完成。落实招商引资项目702项，引进资金90.9亿元，实际利用外资1835万美元。通化被列为国家医药出口基地、全国创业辅导重点城市。通化经济开发区被批准为省级开发区，规划建设全面展开。民营经济增加值增长20.6%、纳税额增长21%。粮食生产和农民收入同步增长。免征农业税综合配套改革扎实推进。向县（市）下放了经济社会管理权限。各县（市）工业集中区建设全面启动。新建通屯水泥路1148公里。绿化乡村水泥路2027公里。防止了禽流感等重大疫情的发生。全省城镇规划建设管理现场会在我市召开。年初确定的城建重点工程全面完成。改造街巷26条、15万平方米。解决了3.5万人吃水和1.2万人用气问题。实施城市“安居工程”，解决市区特困群众住房300户。市青少年宫、广播电视中心、抗日联军纪念馆、通化长白山碑苑、万发遗址博物馆、通化卫校等社会事业重点工程基本完工。成功举办了杨靖宇诞辰100周年纪念活动。靖宇陵园列入全国红色旅游经典景区。平安通化建设取得成效，被评为全国社会治安综合治理先进市。实施了机关工作人员“六不准”规定，查处涉软案件37件。在全省万户企业评政府（行业）活动中，我市平均满意率居各市（州）前列。

　　各位代表，过去的五年，是通化经济和社会事业快速发展的五年，是城市面貌和人居环境明显改善的五年，是人民群众不断得到实惠的五年。这些成绩的取得，是市委正确领导的结果，是市人大、市政协有效监督、大力支持的结果，凝聚着全市人民的辛勤劳动和聪明才智。在此，我代表市政府，向广大干部群众，向人大代表、政协委员，向各民主党派、各人民团体、各界人士，向驻通国省属单位，向驻通部队、武警官兵，向所有关心、支持通化发展的同志们、朋友们，表示崇高的敬意和衷心的感谢！

　　我们也清醒地认识到，我市经济社会发展还存在不少矛盾和问题，主要是经济总量偏小，综合实力不强，经济外向度和市场化水平不高；工业结构调整任务重，产业规模不大，产业链条不完整；交通等基础设施和要素平台的支撑能力不足，开放招商存在诸多制约因素；“三农”问题仍然突出，农民增收相对缓慢；城市文化品位和服务功能仍需提高，社会事业欠账较多；就业和社会保障压力较大，一些群众的生活还有许多实际困难；软环境建设仍有差距，公务员的工作作风、业务水平有待进一步改进和提高，个别工作人员为政不廉，等等。我们一定要高度重视这些问题，认真加以解决。

　　二、“十一五”发展目标和主要任务

　　“十一五”是振兴通化老工业基地和全面建设小康社会的重要战略机遇期。国际经济处于新一轮恢复增长期，国内经济进入消费升级、结构优化的新阶段，将为经济增长创造巨大空间。国家支持东北振兴、南资北移势头强劲、全省加大固定资产投资力度，有利于我市加快发展。但同时，能源、资金等生产要素仍然紧张，钢铁、医药、食品等产业的市场竞争日趋激烈，全国各地竞相发展，对我们提出了新的挑战。我们必须抢抓机遇，真抓实干，使通化在新的起点上更快更好地发展。

　　“十一五”时期，我市经济社会发展总的指导思想是：以邓小平理论和“三个代表”重要思想为指导，全面贯彻落实科学发展观，以振兴通化老工业基地为中心任务，以提高人民生活水平为根本出发点，牢固树立工业立市、工业强市思想，发展壮大医药、冶金、食品三大支柱产业，加快“医药城”、“钢铁城”、“葡萄酒城”建设，大力发展机械制造、能源、新型建材、旅游等优势产业，推进工业化、城镇化和农业现代化进程，促进经济、社会、人口、资源、环境、城乡协调发展，打造活力通化、开放通化、诚信通化、平安通化、和谐通化，为实现更快更好地发展和全面建设小康社会目标奠定更加坚实的基础。

　　推动“十一五”时期更快更好地发展，我们要坚持以科学发展观为统领，把发展作为解决所有问题的关键；转变经济增长方式，增强自主创新能力，努力提高发展的质量和效益；解放思想，开拓创新，牢牢把握机遇，加快改革开放；切实解决关系群众利益的突出问题，推进和谐社会建设；凝聚全市干部群众的积极性、创造性，进一步形成抓发展、抓重点、抓落实的浓厚氛围和强大合力。

　　根据以上指导思想和原则，我市“十一五”发展的主要目标是：地区生产总值年均增长15%，经济总量突破500亿元；财政收入保持同步增长；固定资产投资总规模达到1000亿元；单位GDP能耗降低30%左右；城镇居民人均可支配收入年均增长10%，农民人均纯收入年均增长7%。到2010年，基本完成老工业基地调整改造任务。

　　（一）推动产业结构优化升级，提升产业综合竞争力。突出工业主导地位，进一步发展壮大支柱和优势产业，实现老工业基地向新型产业基地的转变。加大新药开发力度，加速资源整合、企业重组，加强医药产业的服务体系建设，构建生产和商贸服务平台，打造国家级现代中药、生物医药产业基地、医药出口基地和长白山中药材基地。产值超亿元的医药企业发展到20户以上。全力支持通钢实施1000万吨钢发展规划，扩大原辅材料供应，搞好中间产品转化、钢产品深加工，形成配套项目和企业集群，打造吉林省冶金产业基地。到2010年，医药和冶金产业总量实现翻番，分别达到140亿元、230亿元。依托丰富的农特产品资源和生态资源，扶持壮大一批龙头企业，打造长白山绿色食品产业基地。食品产业产值实现翻两番，达到74亿元。葡萄酒营销能力达到12万吨。建成国家级果酒及果蔬饮品检测中心。加快发展优势产业和高新技术产业，形成多元化产业体系。机械制造、能源、新型建材产值达到60亿元，高新技术企业发展到50户以上。大力发展旅游业，创建国家优秀旅游城市。旅游收入达到27亿元。建设区域性现代物流中心，服务业增加值年均增长14%。高起点运作、高标准建设、高效率运行，把通化经济开发区建设成为经济发展的载体、招商引资的窗口、特色产业的集聚区和城市建设的新亮点，成为全市经济的重要支撑。

　　（二）扎实推进社会主义新农村建设，认真解决“三农”问题。按照生产发展、生活宽裕、乡风文明、村容整洁、管理民主的要求，分阶段、有重点地推进新农村建设。突出工业拉动、城乡互动、科技推动，以农村工业化带动农业产业化，实现县域经济快速发展。农业产业化龙头企业加工转化能力和销售收入实现翻番，带动农户60%以上。大力发展现代农业，加快商品粮基地、优质粮产业工程和园艺特产示范区建设。深入实施“粮变肉”工程，做强做大畜牧业。积极发展林地经济和林产品精深加工。把培育新农民与促进劳动力转移结合起来，抓好“一推双带”，建立农民收入持续稳定增长的机制。强化政府对农村的公共服务和科技服务，加强对农民的技能培训。开展新农村建设试点，用五年时间，使全市1/3的行政村达到新农村建设标准。加大农村教育、卫生、文化等投入，促进农村社会事业全面发展。实施农村义务教育经费保障机制，到2007年全市农村中小学生全部免收学杂费，逐步扩大免费教课书范围。完成县乡两级医疗机构和公共卫生体系建设。建立新型农村合作医疗制度。加快推进文化信息资源共享工程和广播电视村村通工程。加强乡村道路、水利设施建设，发展农村通信，完善农村电网。实施好村村通自来水工程，解决农村饮水安全问题。发展县城和区域中心镇，形成特色突出、布局合理的城镇体系。

　　（三）加快基础设施建设，构筑现代化城市框架。全市新增公路通车里程1500公里，其中高速公路建设规模达到508公里。通沈、通丹、通长、梅沈高速公路开工建设，市区与各县（市）基本实现高等级公路连结，县乡公路基本实现区域网络化，形成以“一环五纵八横”为骨架的进关出海快速通道和公路运输网络。新建东北东部铁路，改造通梅铁路。开工建设通化机场。进一步调整优化市区空间布局，建设和完善江西商业区、江南新区、二道江工业区、江北物流区和开发区五大功能分区，形成鸡冠山、玉皇山两个山体景观视廊和浑江、保安河、二道河等景观带。高水平谋划建设一批与城市特色相协调、城市定位相一致的重大城建项目，形成比较发达的市政公用、综合交通、信息通讯、能源供给、水资源保障等基础设施体系，成为吉林省东南部中心城市和更加适宜创业和人居的山水城市。2010年，城市供水普及率达到92.5%，新增供热面积600万平方米，燃气气化率达到80.5%。

　　（四）坚持统筹协调，提高社会事业发展水平。深入实施“科教兴市”战略，努力建设创新型城市。加大科技投入，加强产学研合作，加快科技创新和科技成果产业化。争取20个项目列入国家科技专项。深化教育改革，推进素质教育，优化教育结构，建立现代国民教育体系和终身学习体系。整合职业教育资源，办好高等职业技术学院和职教中心。抓好人才培养工程，培养、引进、用好各类人才。实施文艺精品战略，深化文化体制改革，挖掘优秀文化资源，加快发展文化事业和文化产业。进一步加强文物保护工作。完善社会事业基础设施，规划建设市图书馆、博物馆、体育场馆、滑雪休闲运动中心等重点项目。70%的社区和20%的乡镇建成公益性体育设施。稳定低生育水平，提高人口素质。完善医疗卫生服务体系，提高群众健康水平。

　　（五）深化重点领域改革，全方位扩大对外开放。完成国有经济布局和结构战略性调整，加快农林、供销、物资、商贸流通、建筑施工等行业的改革。社会事业、公共服务等领域的改革取得新的进展。民营经济占经济总量的比重达到70%，对财政的贡献率达到65%。深化行政管理体制改革，进一步减少和规范行政审批，加强经济调节、市场监管、社会管理和公共服务职能。深化财政体制改革，抓好财源建设，建立公共财政框架。加强金融生态环境建设，提高金融对经济社会发展的支撑能力。深化投融资体制改革，建立充满活力的资本运营体系。加快发展土地、技术和劳动力等要素市场。大力发展开放型经济，积极引进战略投资者，注重引进大项目尤其是产业项目。做大支柱产业和优势产业以及长白山特色产品出口基地，把更多的地方产品推向国际市场。“十一五”时期，引进内资400亿元，实际利用外资1亿美元，实现外贸进出口总额18.3亿美元。

　　（六）加强生态建设和环境保护，增强可持续发展能力。实施一批生态保护和环境治理工程、资源节约重点工程、循环经济示范工程，建设资源节约型、环境友好型城市。全市新增林地面积60万亩，森林覆盖率达到65%。实现连续55年无重大森林火灾。继续开展城乡环境综合整治，争创国家级卫生城市、国家园林城市。市区新增绿地面积20万平方米，人均公共绿地面积达到9.1平方米，建成污水处理厂，垃圾无害化处理率达到50%，工业固体废弃物综合利用率达到80%，饮用水源水质达标率95%以上，空气环境质量二级以上天数超过300天。强化对水源、湿地、矿产等资源的规划、保护和利用。

　　（七）贯彻以人为本理念，努力构建和谐通化。统筹城乡就业，加强就业服务，千方百计扩大就业。积极开发公益性就业岗位，重点解决困难群众的就业问题。进一步完善社会保障、社会救助体系，提高城市低保标准，建立农村最低生活保障制度和被征地农民的社会保障办法。完善住房公积金制度。着力提高低收入者收入水平，逐步扩大中等收入者比重。三年拆迁、五年完成市区150万平方米棚户区改造任务，改善12万低收入和特困群众的生活居住条件。落实《公民道德建设实施纲要》，提高全市人民的思想道德素质，争创全国文明城市。开展全民诚信教育，打造诚信政府、诚信企业。完善社区配套设施和服务功能。创新社会管理体制，提高保障公共安全的能力。

　　三、2006年工作安排

　　按照市委四届七次全会的部署，加大工作力度，为顺利实施“十一五”规划开好局、起好步。地区生产总值预期增长15%，财政收入按可比口径同步增长，固定资产投资增长36.4%，规模以上工业增加值增长15%以上、利润增长10%以上，社会消费品零售总额增长13%，城镇登记失业率控制在5%以内，城镇居民人均可支配收入和农民人均纯收入分别增长10%和7%，人口自然增长率控制在2.8‰以内。

　　（一）组织开展“项目建设年”活动，进一步加大招商引资力度。集中广大干部群众的智慧和力量，坚持以项目为中心，通过建设一批大项目、好项目，推动投资和招商引资大幅增长，推动经济快速发展和社会全面进步。把扩大投资的重点放在工业项目和交通、能源、水利、城市基础设施建设上，放在调整结构、转变增长方式和可持续发展上。实施亿元以上重点项目84项，工业投资达到80.7亿元。成立重大项目建设领导小组和重点工作推进组。继续争取国家和省里的项目及资金支持。加强同开发银行、商业银行等金融机构的沟通和对接，建立政府、企业、银行合作机制。加大项目的招商引资力度，大力推进产业招商、园区招商和专业招商，创新方式方法，提高引资实效。重点组织好国外赴欧洲、日韩俄、东南亚，国内赴“长三角”、“珠三角”、“环渤海”等地开展经贸活动，积极引进战略投资者和大企业、大项目。全市引进内资29.7亿元，实际利用外资3300万美元；引进投资1000万元以上的项目200个。强化招商引资工作责任制，从市级领导班子做起，针对不同行业下达项目任务，组建若干招商工作组，采取相应的考核及奖励措施。

　　（二）提高工业增长质量，促进经济平稳运行。全面启动“十一五”百项重点技改项目和百种新产品试产计划，加快冷轧、人胰岛素、葡激酶、热电、桃园变电等重点项目建设进度，振兴通化老工业基地23个工业项目和高技术专项争取多数建成投产。加快发展钢铁衍生经济，积极开发上下游产品及配套项目。加速医药、葡萄酒企业重组，扶持骨干企业做大做强。医药工业产值增长10%，葡萄酒销售收入增长15%。完成通化山葡萄酒原产地域产品保护申报和实施工作。大力发展食品加工、石油机械及汽车零部件制造业，促进产业集群发展。应用信息技术改造提升传统产业。加大油页岩、煤炭、铁矿等资源的勘探开发力度。坚持以规范促发展，加强医药、葡萄酒等行业的质量监管。培育发展名牌产品、著名商标和原产地标识产品，创省级以上名牌产品10个。积极发展电子商务、物流配送、连锁经营等新型流通业，加强市场营销，扩大产品销售。加快医药、钢铁、农特产品出口基地和边境口岸、通道建设，鼓励有条件的企业“走出去”，扩大产品出口及经贸合作。实现外贸进出口总额3亿美元。加强煤电油运等生产要素的衔接和组织，保证重点产业、重点企业的生产运行。开展争创全市优秀工业企业和优秀产品评选活动。抓好节能降耗，发展节约型产业。单位GDP能耗降低6%。依法加强税收征管，确保财政收入稳定增长。

　　（三）加快发展县域经济，启动社会主义新农村规划建设。突出抓好县（市）工业集中区建设，完善基础设施和配套服务，增强产业集聚和资本吸纳能力。推进农业产业化经营，着力发展绿优米、人参、中药材、葡萄、果仁、山野菜、畜禽、林蛙、蜂业、食用菌10个特色产业，培育壮大卓越、正方、中兴、阜康、新开河等38户龙头企业。提高粮食综合生产能力，优质水稻、特用旱粮、经济作物种植面积保持在300万亩以上。实施“十万头基础母牛”工程，建设100个标准化牧业小区，抓好鹅肥肝、肉猪、肉鸡、乳制品等加工项目。积极防控禽流感等重大突发疫病。实施1万亩林下参、6万亩经济林和速生丰产林、100条标准化生态沟建设工程。鼓励发展农村合作经济组织。转移农村劳动力17万人以上。制定新农村建设总体规划，选择70个行政村作为试点，重点搞好公益事业和基础设施建设。实施“清洁文明工程”，深入开展农村环境综合整治。抓好江河治理、水库除险加固等水利设施建设。新建农村水泥路300公里，建成农村人畜饮水工程20处。稳步推进农村综合改革。进行农业普查。

　　（四）全力推进通化经济开发区建设，打造新的发展平台。坚持统一规划、分步实施、滚动发展的原则，举全市之力、集各类要素，加快开发区建设，使之成为引领通化经济快跑的“火车头”。突出抓好冶金、机械制造、生物医药、有机食品4个工业园区规划建设，科学合理地确定功能分区，逐步形成多园并举、各具特色、集群发展的格局。积极争取政策性资金和软贷款，搞好多元化、市场化融资，重点建设张家园区等基础设施。加大开发区招商引资，广泛吸引域外大企业、大项目入驻园区。引进战略投资者合作开发建设机械、汽车零部件工业园区。全市的产业布局和引进的项目，重点向开发区倾斜。鼓励东昌、二道江区和通化县向开发区招项目、摆项目，实行产值分计、税收分享。结合产业结构调整、技术改造，支持有条件的市区工业企业实施置换、重组，向开发区集聚。

　　（五）继续深化国企改革，大力发展民营经济。抓好国企改革攻坚的收尾工作，切实解决遗留问题。把改制和发展紧密结合起来，加强规范和管理，增强改制企业发展活力。基本完成商贸、交通、建筑和林业等非工领域的国企改革任务，积极推进国有公益性改革试点。加强对股份制企业的监管，完善公司法人治理结构，促其规范运行、健康发展。加强产权交易平台、信用担保中心和中小企业孵化器建设。启动新一轮上市融资预案。完善创业大厦服务功能，推动民营经济快速发展。民营经济增加值增长20%、纳税额增长15%。整合旅游资源，加强服务设施建设，大力开发特色旅游项目。做好靖宇陵园、白鸡腰、五女峰4A级景区申报工作。接待海内外游客和旅游收入分别增长27%。加快发展通化、梅河口物流园区，培育和引进一批物流骨干企业。探索建设长白山药材、药品流通市场，做大汽车交易和建材、人参、蔬菜等特色批发市场。稳步发展房地产业，搞活住房二、三级市场。

　　（六）加强城市建设和管理，提升承载能力和服务功能。搞好城市总体规划修编，高标准编制分区规划和控制性详规。完成新一轮土地利用总体规划修编，努力实现城区土地收益最大化。市区绕越线公路竣工通车，通沈高速公路完成路基、桥涵工程量的50%。做好通长、通丹高速公路和东北东部铁路、通化机场等重大项目的前期工作。坚持全面规划、合理布局、因地制宜、综合开发、配套建设，全力打好棚户区改造攻坚战，完成50万平方米拆迁改造任务，确保开发建设质量和拆迁户回迁安置。加快江南新区路网建设，形成局部道路骨架。对滨江西路红旗桥至江北大桥段进行综合改造，改扩建江南大桥北端立交桥，改造龙泉路、新风路等12条标准路和巷路。改造供水设施，解决市区周边及高层4.2万人吃水问题。实施第二煤气站一期工程，解决3000户居民用气问题。继续改造老旧楼供热设施。搞好城区绿化彩化，解决好巷路照明问题。深入推进城区环境综合整治，抓好卫生保洁、周山沟河及城乡结合部整治，完善市容市貌、环境卫生、物业等方面的长效管理机制。实施交通畅通工程，加快建设停车场。加强污染防治，整顿、规范矿产资源开发秩序。加大哈泥河湿地和饮用水源的环境测评和保护力度。

　　（七）加快社会事业发展，加强精神文明建设。重点实施一批振兴老工业基地科技发展专项，加强原始创新、集成创新和引进消化吸收创新。发展基础教育、职业教育，全面实施素质教育。实现100所学校宽带联通。启动农村初中标准化建设工程，改造农村危旧校舍3万平方米。严格实行义务教育阶段“一费制”，抓好校园安全和周边环境整治。支持通化师院加快发展。市青少年宫、广播电视中心交付使用。积极推进10个人才重点项目和事业单位人事制度改革。繁荣文艺创作，丰富群众文化生活。举办首届文化艺术节。加大对《英语辅导报》等文化产业的扶持力度。实施高句丽遗迹保护二期工程，做好第六批国保、省保单位申报工作。深化城市医疗服务体制改革，启动第二批新型农村合作医疗试点。完成市医院门诊和通化卫校配套工程建设，抓好中心医院外科大楼建设工程。落实全民健身计划，备战省运会和全国冬运会，争取好成绩。建成体育新村，加快滑雪场建设。做好通化数字电视整体平移的推动工作。全面推行计划生育奖励扶助政策。广泛开展文明城市、文明行业、文明村镇、文明社区、文明家庭创建活动。发展社区经济，搞好社区建设。维护妇女儿童、老年人、残疾人合法权益。深入开展好军（警）民共建活动，争创全国双拥模范城“六连冠”。

　　（八）认真解决关系民生的问题，维护社会和谐稳定。落实再就业优惠政策，促进稳定就业。全市开发就业岗位5.6万个，新增就业4.5万人。继续扩大社会保障覆盖面，探索事业单位养老保险制度改革，规范和完善城乡低保制度。新增养老、失业、医疗、工伤、生育保险参保人数9万人。推进被征地农民的社会保障工作。加强社会救助体系建设，着力解决困难群众的住房、大病就医、子女上学等实际困难。继续实施联乡包村万户脱贫工程，使100个村、1万户农民基本实现脱贫。重视抓好信访和市长公开电话工作，依法解决与群众利益相关的问题。整顿和规范市场秩序，强化食品、药品和餐饮卫生等监管，保障人民群众健康安全。全面落实安全生产责任制，加大监督检查力度，严防重特大事故发生。加强社会治安综合治理，严厉打击各类犯罪，建设平安通化。加强防灾救灾体系建设。建立完善突发事件预警和应急机制。合力推进“五边”试点，维护边境安全稳定。

　　（九）坚持依法行政，建设服务型政府。自觉接受人大的依法监督、政协的民主监督。规范行政执法行为，公正执法，文明执法。组织开展“五五”普法。进一步改善经济发展软环境，为企业和投资者提供优先、优惠、优质服务和全天候、全方位、全过程服务。建立决策智力支撑体系，科学决策、依法决策、民主决策。落实新闻发布和重大决策、重要事项公开咨询、公开听证和公示等制度，全面推行公用事业单位公开办事制度。进行政府应急信息管理平台和网上业务系统建设，加快发展电子政务。完善行政审批中心服务功能。开展全民诚信教育，营造良好的信用环境。认真贯彻《公务员法》，努力建设高素质的公务员队伍。弘扬求真务实和创新精神，大力改进政风，狠抓工作落实，提高行政效能。政府服务要走出机关、重心下沉，积极主动地为基层和群众解决实际问题。构建预防和惩治腐败体系，强化行政监察、审计监督，坚决查处违法违纪案件。各级公务员要始终铭记人民的重托，牢固树立以人为本、执政为民的理念，心系群众，恪尽职守，干净干事。

　　各位代表！回首过去的五年，成就鼓舞人心；展望未来的发展，任务催人奋进。让我们在市委的坚强领导下，在市人大、市政协的大力支持下，团结一致，开拓进取，为实现“十一五”规划目标、为振兴通化老工业基地而努力奋斗！
